# Supplementary material for: Content-rich biological network constructed by mining PubMed abstracts
Source: BMC Bioinformatics. 2004 Oct 8;5:147. doi: 10.1186/1471-2105-5-147 (PMC528731; doi:10.1186/1471-2105-5-147)
Supplement: Additional File 2 — The original results of the above study (non-essential files are deleted to keep the file size under the limit set by BMC bioinformatics). [file 1471-2105-5-147-S2.bz2 › chilibotAdditionalFile2/dip40/389ID11297552E9864/html/PDGFRB_PDGFB.html]

 


 **PDGFRB** and **PDGFB** 
  
Found 434 abstracts in PubMed, retrieved 40.  
 

 What does Google say? 
 PDF only 
| .edu only 

---

**Interactive relationship** (e.g. stimulation, inhibition, etc)

**Neutral relationship**- The platelet derived growth factor  [ **PDGFB** ]  PDGF B PDGF receptor PDGFR  [ **PDGFRB** ]  beta system, hitherto thought to contribute mainly to neuroprotection, may also support angiogenesis and vascular remodeling by mediating interactions of endothelial cells with pericytes after cerebral ischemia.  Ref: 12750005 Brain Res Mol Brain Res, 2003
- PDGF B, the cellular homologue of the viral simian sarcoma oncogene v sis  [ **PDGFB** ] , is known to potently induce cellular transformation through activation of PDGF receptor PDGFR  [ **PDGFRB** ]  beta.  Ref: 12629513 Oncogene, 2003

**Non-interactive relationship** (e.g. studied together, co-existance, homology, etc.)

- We previously reported that the platelet derived growth factor B chain  [ **PDGFB** ]  PDGF B PDGF receptor PDGFR  [ **PDGFRB** ]  axis is involved in tubular regeneration after ischemia reperfusion injury of the kidney.  Ref: 12819032 Am J Pathol, 2003
- Time and cell type specific induction of platelet derived growth factor  [ **PDGFB** ]  receptor beta  [ **PDGFRB** ]  during cerebral ischemia.  Ref: 12750005 Brain Res Mol Brain Res, 2003
- Sodium hydrogen exchanger regulatory factor 1 and 2 NHERF 1 and NHERF 2 are adaptor proteins that regulate renal electrolyte transport and interact with the platelet derived growth factor  [ **PDGFB** ]  receptors PDGFR  [ **PDGFRB** ] .  Ref: 12865627 PathobiologyPathobiology, 2003
- We also showed that the A549 lung cancer cells expresses the platelet derived growth factor  [ **PDGFB** ]  receptor alpha, and the inhibitory effects of Gleevec on A549 cells is likely mediated through inhibition of PDGFR  [ **PDGFRB** ]  alpha phosphorylation.  Ref: 12537587 Mol Cancer< MedlineTA>Mol Cancer, 2003
- mRNAs of platelet derived growth factor  [ **PDGFB** ]  A PDGF A and PDGF receptor PDGFR  [ **PDGFRB** ]  alpha were markedly up regulated in the skin during spontaneous and thyroid hormone TH induced metamorphosis.  Ref: 12761844 Dev Dyn, 2003
- Effect of ribozyme against platelet derived growth factor  [ **PDGFB** ]  receptor beta  [ **PDGFRB** ]  subunit mRNA on the biological characters of hepatic stellate cells < ArticleTitle>.  Ref: 12773241 Zhonghua Gan Zang Bing Za Zhi, 2003
- Platelet derived growth factor  [ **PDGFB** ]  receptor beta  [ **PDGFRB** ]  like tumor suppressor gene PRLTS and deletion in liver cancer 1 tumor suppressor gene are located at 8p21.3 p22.  Ref: 12653892 J Gastroenterol Hepatol, 2003
- Novel translocations that disrupt the platelet derived growth factor  [ **PDGFB** ]  receptor beta  [ **PDGFRB** ]   **PDGFRB**  gene in BCR ABL negative chronic myeloproliferative disorders.  Ref: 12542482 Br J Haematol, 2003
- Immunohistochemical procedures were performed to detect the following protein antigens CD99, interleukin IL 1alpha, IL 6, transforming growth factor TGF alpha, conventional c protein kinase C cPKC alpha, cPKC betaII, phosphorylated p PKC alpha betaII, c kit CD117, platelet derived growth factor  [ **PDGFB** ]  receptor PDGFR  [ **PDGFRB** ]  alpha, PDGFR  [ **PDGFRB** ]  beta, epidermal growth factor receptor EGFR, human epidermal growth factor receptor HER 2 neu, cathepsin D, angiotensin converting enzyme ACE, angiotensin II type 1 AT1 receptor, p21ras, the alpha subunit of farnesyl and geranylgeranyl transferase FTalpha GGTalpha, phospho p c Jun N terminal kinase p JNK, p p38 mitogen activated protein kinase MAPK, cyclin D1, c Jun, Ki 67, bcl 2, TGF beta1 latency associated peptide LAP, TGF betaRII, and cyclooxygenase COX 2.  Ref: 12817616 Ann Clin Lab Sci< MedlineTA>Ann Clin Lab Sci, 2003
- Expression of platelet derived growth factor  [ **PDGFB** ]  receptor beta  [ **PDGFRB** ]  in fibroblasts of keloid < ArticleTitle>.  Ref: 12516475 Zhongguo Xiu Fu Chong Jian Wai Ke Za Zhi, 2000
- Recent reports of responses to imatinib in patients with the syndrome suggested that an activated kinase such as ABL, platelet derived growth factor  [ **PDGFB** ]  receptor PDGFR  [ **PDGFRB** ] , or KIT, all of which are inhibited by imatinib, might be the cause.  Ref: 12660384 N Engl J Med, 2003
- cKit and platelet derived growth factor  [ **PDGFB** ]  receptor PDGFR  [ **PDGFRB** ]  are receptor tyrosine kinases expressed in the testis, are involved in testosterone production, and are inhibited by imatinib.  Ref: 12801741 Lancet, 2003
- The proto oncogene c kit is a transmembrane tyrosine kinase receptor that is structurally related to the platelet derived growth factor  [ **PDGFB** ]  receptor PDGFR  [ **PDGFRB** ]  and is involved in cell differentiation.  Ref: 12649669 Int J Gynecol Pathol, 2003
- This review describes the preclinical and clinical status of low molecular weight drugs targeted against different tyrosine kinases e.g., epidermal growth factor receptor EGFR, vascular endothelial growth factor receptor VEGFR, platelet derived growth factor  [ **PDGFB** ]  receptor PDGFR  [ **PDGFRB** ] , Kit, Fms like tyrosine kinase Flt 3, briefly describes new targets, and provides a critical analysis of the current situation in the area of tyrosine kinase inhibitors.  Ref: 12667099 Expert Opin Ther Targets, 2003
- A possible regulation of this transport system by platelet derived growth factor  [ **PDGFB** ]  receptor PDGFr  [ **PDGFRB** ]  with tyrosine kinase activity is also demonstrated.  Ref: 12822894 IUBMB Life, 2003
- Imatinib Gleevec formerly STI571 is an orally bioavailable rationally developed inhibitor of the tyrosine kinases Bcr Abl, Kit, and platelet derived growth factor  [ **PDGFB** ]  receptor PDGFR  [ **PDGFRB** ] .  Ref: 12783370 Semin Hematol, 2003
- OBJECTIVE To study the cleavage activity of hammerhead ribozyme targeting at platelet derived growth factor  [ **PDGFB** ]  receptor beta  [ **PDGFRB** ]  subunit PDGFR  [ **PDGFRB** ]  beta mRNA in hepatic stellate cells HSCs and its effect on the biological characters of HSCs.  Ref: 12773241 Zhonghua Gan Zang Bing Za Zhi, 2003
- To clarify the mechanisms underlying radiation pneumonitis and pulmonary fibrosis, we investigated the expression of platelet derived growth factor  [ **PDGFB** ]  receptor PDGFR  [ **PDGFRB** ]  on fibroblasts obtained from irradiated rat lungs and on control fibroblasts.  Ref: 12751964 Radiat Res, 2003
- The expression of platelet derived growth factor  [ **PDGFB** ]  PDGF BB and PDGF receptor beta PDGFR  [ **PDGFRB** ]  beta in liver tissues from viral hepatitis was detected by immunohistochemistry.  Ref: 12515172 Zhonghua Shi Yan He Lin Chuang Bing Du Xue Za Zhi, 1998
- Because activation of platelet derived growth factor  [ **PDGFB** ]  PDGF receptor tyrosine kinase PDGFr  [ **PDGFRB** ]  TK influences these processes and promotes restenosis, TKI963, an inhibitor of the PDGFr  [ **PDGFRB** ]  TK was discovered, and its efficacy was evaluated in blocking stent induced restenosis as analyzed by intravascular ultrasound IVUS.  Ref: 12775958 J Cardiovasc Pharmacol, 2003
- In TSC2 expressing VSMCs, growth inhibition in response to E2 was associated with downregulation of platelet derived growth factor  [ **PDGFB** ]  PDGF, platelet derived growth factor  [ **PDGFB** ]  receptor PDGFR  [ **PDGFRB** ]  and limited activation of ERK.  Ref: 12700139 Am J Physiol Cell Physiol< MedlineTA>Am J Physiol Cell Physiol, 2003
- The IPLB LdFB cell line from the fat body of the insect Lymantria dispar shows the presence of immunoreactive, platelet derived growth factor  [ **PDGFB** ]  PDGF AB and transforming growth factor TGF beta1 molecules, as well as the corresponding plasma membrane like receptors, i.e. PDGFR  [ **PDGFRB** ]  alpha, PDGFR  [ **PDGFRB** ]  beta and TGFR beta type II.  Ref: 12770261 J Insect Physiol< MedlineTA>J Insect Physiol, 2003
- In order to study if epidermis may be genetically transformed to a platelet derived growth factor  [ **PDGFB** ]  sensitive compartment we aimed to introduce the gene encoding human platelet derived growth factor  [ **PDGFB** ]  receptor beta  [ **PDGFRB** ]  PDGF beta R into epidermal keratinocytes using a retrovirus derived vector.  Ref: 12713575 J Invest Dermatol, 2003
- SU11248 Inhibits KIT and Platelet derived Growth Factor  [ **PDGFB** ]  Receptor beta  [ **PDGFRB** ]  in Preclinical Models of Human Small Cell Lung Cancer.  Ref: 12748309 Mol Cancer Ther, 2003
- OnM treatment does not affect gp130 expression levels and desensitizes signaling mediated by a transduced chimeric receptor containing extracellular domains of platelet derived growth factor  [ **PDGFB** ]  receptor beta  [ **PDGFRB** ]  PDGFRbeta and the cytoplasmic region of gp130.  Ref: 12724316 J Biol Chem< MedlineTA>J Biol Chem, 2003
- While platelet derived growth factor  [ **PDGFB** ]  PDGF B and its receptor PDGFR  [ **PDGFRB** ]  beta are essential factors for the recruitment of pericytes to brain capillaries during embryonic development, their role in blood vessel maturation during cerebral ischemia is not clear.  Ref: 12750005 Brain Res Mol Brain Res, 2003
- Recently, tumors with platelet derived growth factor  [ **PDGFB** ]  receptor PDGFR  [ **PDGFRB** ]  a mutations have been described in GISTs with wild type KIT.  Ref: 12781070 Curr Oncol Rep, 2003
- The c Abl tyrosine Tyr kinase is activated after platelet derived growth factor  [ **PDGFB** ]  receptor PDGFR  [ **PDGFRB** ]  stimulation in a manner that is partially dependent on Src kinase activity.  Ref: 12652307 Nat Cell Biol, 2003
- We examined the effects of maternal exposure to estrogens on platelet derived growth factor  [ **PDGFB** ]  PDGF receptor PDGFR  [ **PDGFRB** ]  expression in newborn rat testis.  Ref: 12604637 Biol Reprod, 2003
- Activities of angiotensin converting enzyme ACE and aorta phospholipase C PLC were tested, and levels of platelet derived growth factor  [ **PDGFB** ]  receptor beta  [ **PDGFRB** ]  PDGFR  [ **PDGFRB** ]  beta and Galphaq 11 protein were measured by Western blot analysis.  Ref: 12589410 Sheng Li Xue Bao, 2001
- Translocations that disrupt and constitutively activate the platelet derived growth factor  [ **PDGFB** ]  receptor beta  [ **PDGFRB** ]   **PDGFRB**  gene at chromosome band 5q33 have been described in some patients, the most common being the t 5 ; 12 q33 ; p13.  Ref: 12542482 Br J Haematol, 2003
- SU11248 is an oral, multitargeted tyrosine kinase inhibitor with direct antitumor and antiangiogenic activity through targeting platelet derived growth factor  [ **PDGFB** ]  receptor PDGFR  [ **PDGFRB** ] , vascular endothelial growth factor receptor, KIT, and FLT3 receptors.  Ref: 12748309 Mol Cancer Ther, 2003
- Platelet derived growth factor  [ **PDGFB** ]  PDGF B and its receptor PDGF R beta  [ **PDGFRB** ]  are overexpressed in human gliomas and responsible for recruiting peri endothelial cells to vessels.  Ref: 12651601 Am J Pathol, 2003
- BACKGROUND AND OBJECTIVES Chronic myelomonocytic leukemia CMML is a myelodysplastic syndrome that has been associated with the expression of platelet derived growth factor  [ **PDGFB** ]  b receptor  **PDGFRb**  fusion proteins, namely TEL  **PDGFRb** .  Ref: 12681968 Haematologica, 2003
- OBJECTIVE To investigate the expression and distribution of platelet derived growth factor  [ **PDGFB** ]  receptor beta  [ **PDGFRB** ]  PDGFR  [ **PDGFRB** ]  beta in normal skin and keloid and to discuss its biological function in keloid formation.  Ref: 12516475 Zhongguo Xiu Fu Chong Jian Wai Ke Za Zhi, 2000
